# Supplementary figures and images for: DNA Barcoding of the Genus Magnisudis (Aulopiformes: Paralepididae) with a Coastal Record and Biological Features of Magnisudis atlantica
Source: Biology (Basel). 2023 Feb 22;12(3):349. doi: 10.3390/biology12030349 (PMC10045843; doi:10.3390/biology12030349)

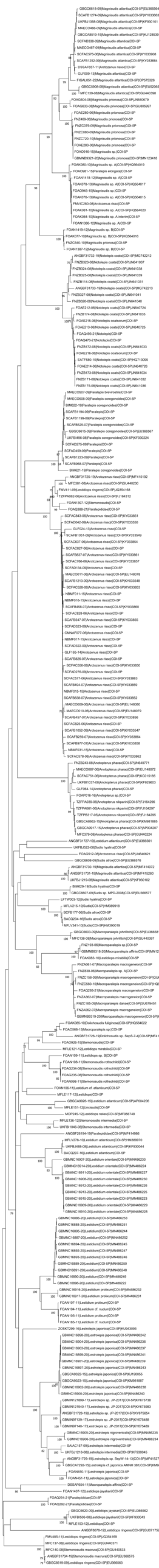

Supplement: Supplementary file 1 [file biology-12-00349-s001.zip › biology-2136465-supplementary.pdf]
